# Ileo-Ileal Knotting Presenting as Strangulated Small Bowel Obstruction: An Uncommon Surgical Catastrophe

**DOI:** 10.7759/cureus.109462

**Published:** 2026-05-22

**Authors:** Aditya Sharma, Nirmal Raj K, Amulya Kanmathareddy, Tanveer Gupta, Sudhir K Singh

**Affiliations:** 1 Department of Surgery, All India Institute of Medical Sciences (AIIMS), Rishikesh, IND

**Keywords:** closed-loop bowel strangulation, ileo-ileal knotting, rare case of intestinal obstruction, small-bowel obstruction, surgical emergency

## Abstract

Ileo-ileal knotting is an exceptionally rare cause of small bowel obstruction in which two ileal loops twist around each other, resulting in vascular compromise and bowel strangulation. Preoperative diagnosis is difficult because of non-specific clinical and radiological findings, and most cases are diagnosed intraoperatively. Delay in treatment may rapidly progress to bowel ischemia and gangrene. We report the case of a 78-year-old male patient who presented with acute intestinal obstruction. Contrast-enhanced computed tomography suggested closed-loop small bowel obstruction without definitive evidence of intestinal knotting. Emergency exploratory laparotomy revealed ileo-ileal knotting with gangrene involving approximately three feet of terminal ileum. Resection of the gangrenous bowel with double-barrel ileostomy was performed successfully. This case highlights the importance of early surgical exploration in rapidly progressive bowel obstruction and emphasizes intestinal knotting as a rare but important differential diagnosis of strangulated obstruction.

## Introduction

Acute intestinal obstruction is a common surgical emergency, with small bowel obstruction accounting for a substantial proportion of cases worldwide [1]. Common etiologies include postoperative adhesions, hernias, malignancy, inflammatory bowel disease, and volvulus. Intestinal knotting is an unusual cause of obstruction in which one bowel loop wraps around another, leading to closed-loop obstruction and vascular compromise [2].

Different forms of intestinal knotting have been described, including ileosigmoid, ileocecal, appendico-ileal, and ileo-ileal knotting. Among these, ileo-ileal knotting is extremely rare [3]. Because of its rapid progression toward ischemia and gangrene, early diagnosis and urgent surgical intervention are essential. However, the absence of characteristic clinical or imaging findings often makes preoperative diagnosis difficult [4].

Although intestinal knotting has been described in several anatomical variants, isolated ileo-ileal knotting remains exceptionally uncommon, with only a limited number of cases reported in the literature [2,3]. Its non-specific clinical and radiological presentation often delays diagnosis until laparotomy, by which time bowel ischemia or gangrene may already be established [4]. Documentation of such rare presentations is important to improve awareness among emergency surgeons and radiologists regarding early recognition, operative decision-making, and the potential need for bowel diversion in elderly patients with strangulated obstruction.

## Case presentation

A 78-year-old male patient presented to our emergency department in March 2026 with complaints of sudden-onset diffuse abdominal pain, progressive abdominal distension, and multiple episodes of bilious vomiting for approximately 12 hours. The abdominal pain was severe, continuous, non-radiating, and progressively worsening in intensity. The patient also reported non-passage of flatus and stools since symptom onset. There was no history of fever, hematemesis, melena, per-rectal bleeding, or recent change in bowel habits. His medical history was significant for chronic obstructive pulmonary disease managed conservatively and chronic tobacco smoking. There was no prior history of abdominal surgery or any other comorbidities.

On initial evaluation, the patient was conscious and oriented. He was hemodynamically stable with a pulse rate of 104 beats/minute (reference range: 60-100 beats/minute), blood pressure of 128/76 mmHg (reference range: systolic 90-120 mmHg; diastolic 60-80 mmHg), respiratory rate of 22 breaths/minute (reference range: 12-20 breaths/minute), and oxygen saturation of 95% on room air (reference range: >94%). He was afebrile with a temperature of 36.8°C (reference range: 36.1°C-37.2°C). General physical examination revealed mild dehydration without pallor, icterus, cyanosis, clubbing, or peripheral edema. Abdominal examination demonstrated generalized abdominal distension with diffuse tenderness, maximal over the periumbilical region. Guarding was present, suggesting evolving peritoneal irritation. No palpable abdominal mass or external hernia was identified. Bowel sounds were absent on auscultation. Digital rectal examination revealed an empty rectum without evidence of blood or intraluminal mass.

**Figure 1:**
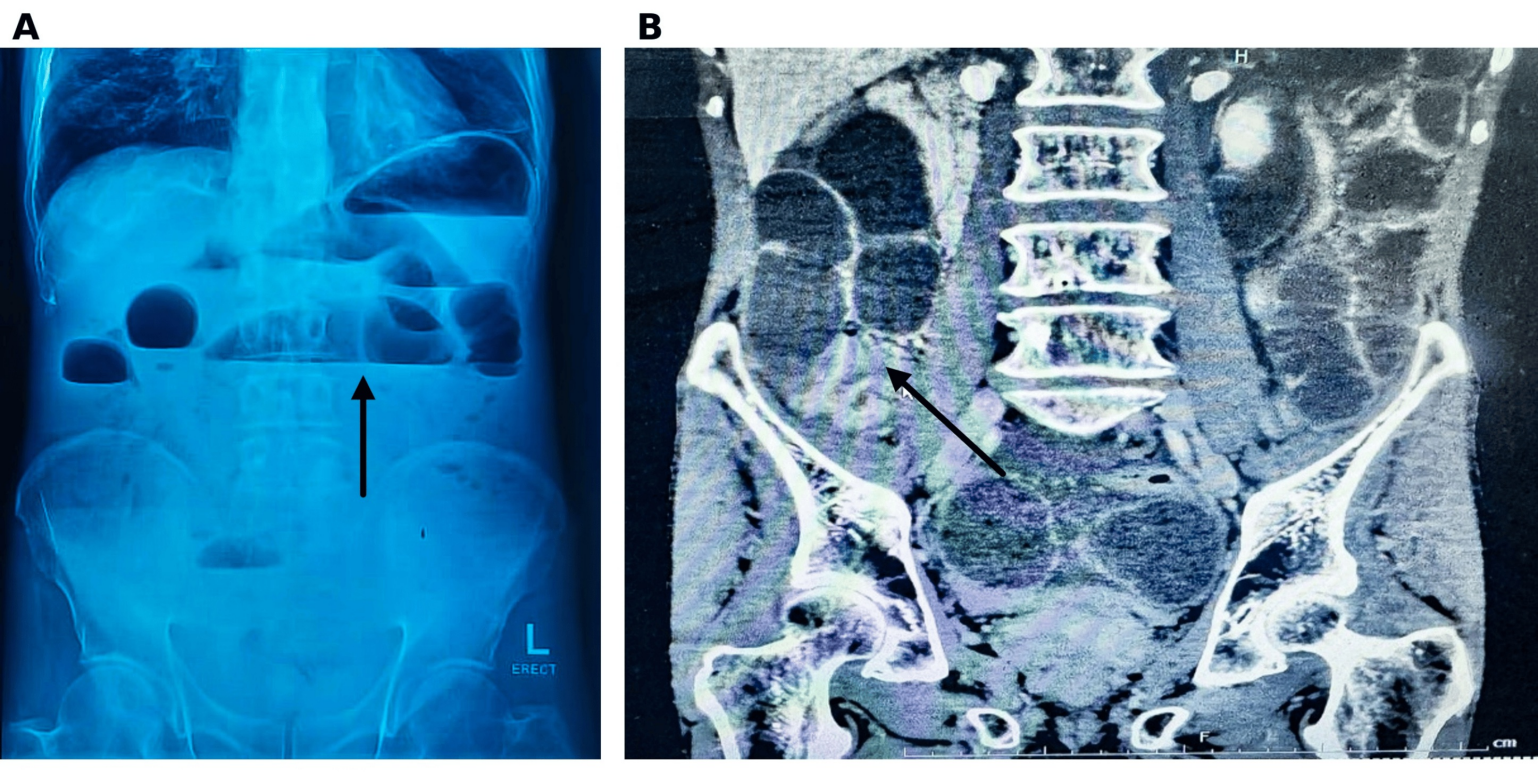
(A) Upright abdominal radiograph demonstrating multiple air-fluid levels; (B) contrast-enhanced computed tomography (CT) showing dilated bowel loops with a transition point suggestive of closed-loop obstruction.

Laboratory investigations revealed leukocytosis with a total leukocyte count of 18.0 × 10⁹/L (reference range: 4.0-11.0 × 10⁹/L) and elevated serum lactate levels of 2.1 mmol/L (reference range: 0.5-2.0 mmol/L). An upright abdominal radiograph showed multiple air-fluid levels suggestive of small bowel obstruction. Contrast-enhanced computed tomography (CECT) demonstrated dilated distal jejunal and proximal ileal loops displaying standard fluid attenuation density (10-20 Hounsfield units (HU)) with multiple air-fluid levels. A distinct transition point was noted in the mid-ileal region, strongly suggestive of a closed-loop obstruction. Notably, the compromised bowel segments demonstrated a subtle decrease in wall enhancement density (30-40 HU) compared to healthy loops (>60 HU), accompanied by surrounding mesenteric fat stranding and fluid density accumulation in the pelvis (~15 HU), which collectively raised suspicion for early ischemic compromise (Figure 1).

**Figure 2:**
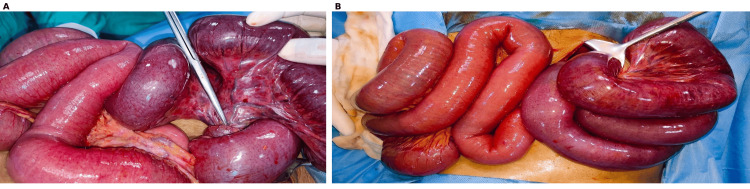
(A) Intraoperative image demonstrating ileo-ileal knotting (instrument pointing); (B) healthy and gangrenous bowel segments following release of the knot.

Based on the acute presentation and imaging findings, differential diagnoses included adhesive small bowel obstruction, internal herniation, closed-loop obstruction secondary to volvulus, mesenteric ischemia, and strangulated obstructed inguinal hernia. However, the absence of previous abdominal surgery and the presence of peritoneal signs increased suspicion for bowel strangulation, requiring urgent operative exploration. Intraoperatively, approximately three feet of gangrenous terminal ileum located nearly one foot proximal to the ileocecal junction was identified. The involved bowel loops were tightly twisted around each other, forming a true ileo-ileal knot with associated mesenteric vascular compromise. No congenital bands, adhesions, diverticula, or internal hernias were identified intraoperatively (Figure 2).

**Figure 3:**
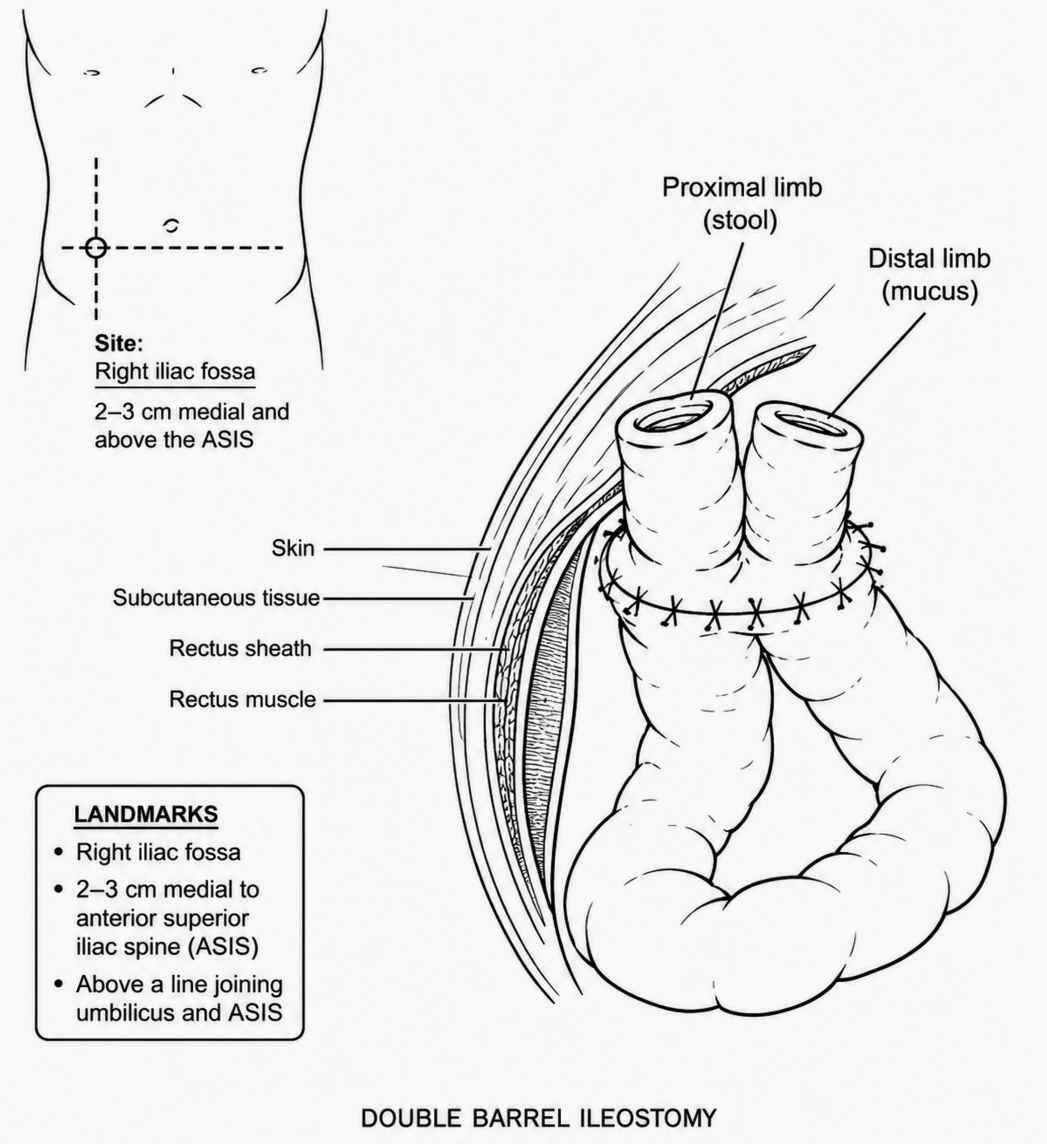
Schematic representation of a double-barrel ileostomy showing the surgical orientation and surface anatomical landmarks. This figure is manually created and labeled by the authors using Adobe Photoshop 2024 (version 25.x; Adobe Inc., San Jose, CA, USA) as an original schematic educational illustration.

The gangrenous bowel was resected after careful release of the knot, and a double-barrel ileostomy was created, considering the patient’s age, bowel condition, and emergency presentation (Figure 3).

Postoperatively, the patient was managed in the high-dependency unit. Transient, expected minor postoperative complications occurred, including mild adynamic ileus-induced vomiting and acute urinary retention, both of which resolved completely with conservative management (nasogastric decompression and temporary urinary catheterization). The patient’s stoma became functional on postoperative day 2. He was successfully transitioned to a solid diet, achieved satisfactory functional status, and was discharged in stable condition. He is currently being followed in the outpatient clinic for nutritional optimization, with an elective, planned reversal of the double-barrel ileostomy scheduled at the 12-week postoperative mark.

## Discussion

Intestinal knotting represents a rare form of closed-loop bowel obstruction associated with rapid vascular compromise and a high risk of bowel gangrene. Among its various subtypes, ileo-ileal knotting is exceptionally uncommon, with very few cases reported in the literature. Similar to previously published reports, our patient presented with rapidly progressive abdominal pain, vomiting, abdominal distension, and radiological evidence of small bowel obstruction. However, preoperative diagnosis remained difficult because imaging findings were non-specific and failed to demonstrate a definitive knot configuration [2,3].

When compared with the sparse existing literature, our case demonstrates both notable similarities and important clinical differences. Most documented intestinal knotting cases involve an ileosigmoid configuration in which a hypermobile ileal segment wraps around a redundant sigmoid colon. In contrast, pure ileo-ileal knotting, as observed in our patient, is considerably rarer because two separate small bowel loops alone form both the axis and the knot. This anatomical distinction likely contributes to the diagnostic difficulty and limited preoperative recognition reported in the literature [4,5].

Most reported cases of ileo-ileal knotting involve younger or middle-aged individuals, often associated with sudden ingestion of bulky, high-fiber meals leading to forceful, asymmetrical peristalsis and excessive bowel mobility [2,4]. Our patient differed significantly from this demographic profile, as he was a 78-year-old elderly man with a virgin abdomen and no identifiable dietary precipitating factors. Advanced age may have contributed to altered bowel motility, age-related mesenteric and visceral anatomical changes, and increased bowel mobility, thereby predisposing to spontaneous knot formation. Intraoperatively, no adhesions, congenital bands, diverticula, or internal hernias were identified, further supporting the possibility of spontaneous ileo-ileal knotting related to excessive bowel mobility rather than secondary mechanical pathology.

**Figure 4:**
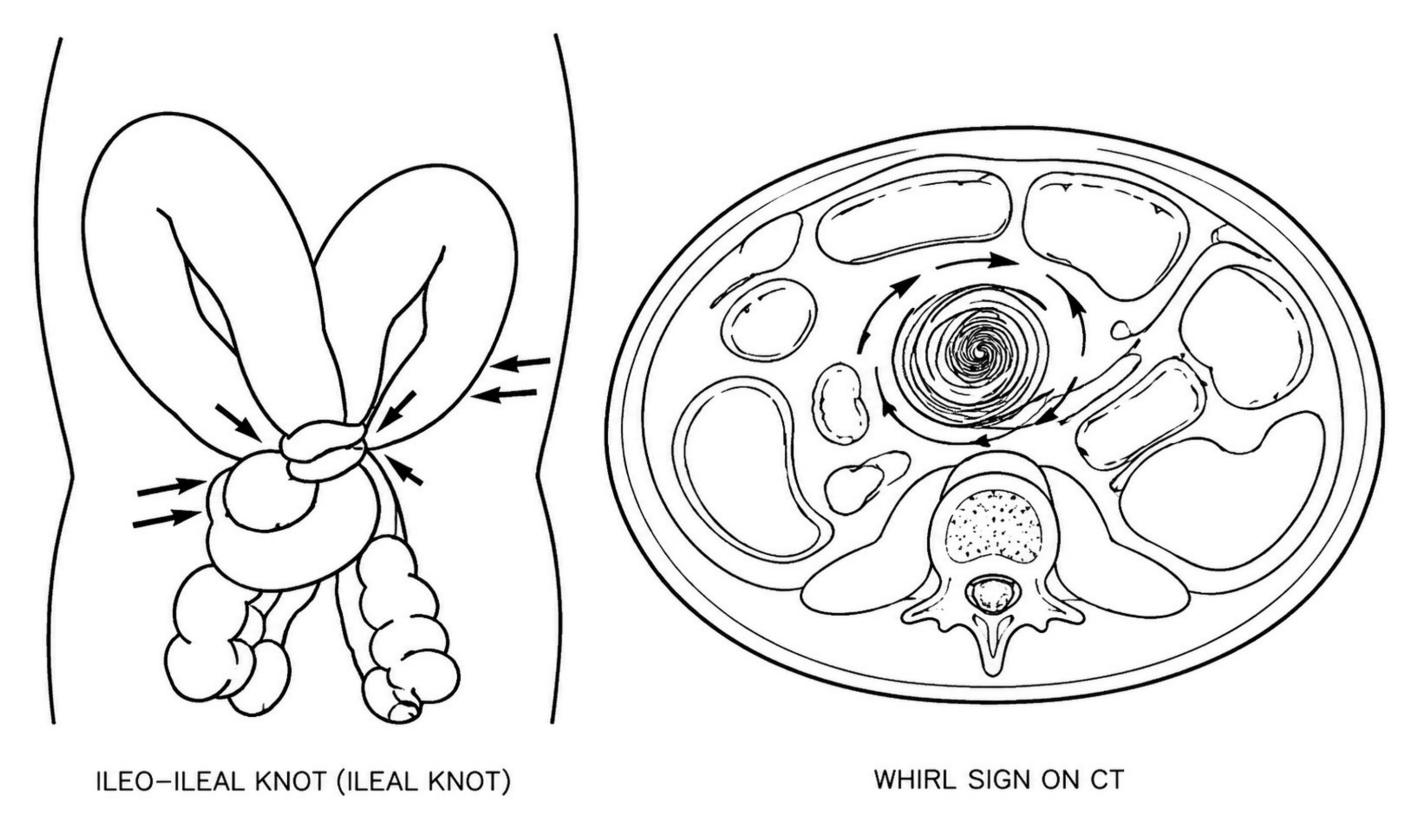
Schematic representation of ileo-ileal knotting and the characteristic “whirl sign” on computed tomography (CT). This figure is manually created and labeled by the authors using Adobe Photoshop 2024 (version 25.x; Adobe Inc., San Jose, CA, USA) as an original schematic educational illustration.

Similar to the cases reported by Taniguchi et al. and Abebe et al., gangrenous bowel was already present at the time of laparotomy, emphasizing the aggressive nature of vascular compromise in intestinal knotting [1,3]. In contrast, Mohammed et al. described a viable ileo-ileal knot managed successfully without bowel resection, likely reflecting earlier diagnosis and surgical intervention before irreversible ischemia developed [2]. These observations collectively suggest that the timing of operative intervention remains the most important determinant of bowel salvage and overall outcome. Radiological diagnosis remains particularly challenging. Although the characteristic “whirl sign” has been described in intestinal knotting and volvulus, it is inconsistently visualized and may not always be evident on preoperative imaging (Figure 4) [6,7].

In our patient, CECT demonstrated closed-loop obstruction with reduced mural enhancement, bowel wall thickening, and mesenteric edema suggestive of strangulation; however, a definitive knot configuration could not be identified. This finding mirrors most previously documented reports in which definitive diagnosis was established only during exploratory laparotomy. The present case, therefore, reinforces the practical surgical principle that in patients with rapidly progressive obstruction and clinical evidence of peritonitis or ischemia, operative decision-making should not be delayed solely because of inconclusive imaging findings.

Surgical management primarily depends on bowel viability and the physiological status of the patient. While viable bowel may occasionally be managed with careful untwisting alone, gangrenous bowel requires urgent resection to prevent perforation, sepsis, and mortality [8]. In the present case, approximately three feet of terminal ileum were gangrenous at exploration. Considering the patient’s advanced age, bowel edema, emergency presentation, and risk of anastomotic complications, resection with double-barrel ileostomy was considered safer than primary anastomosis. This approach minimized the risk of postoperative anastomotic leak and facilitated safer postoperative recovery.

Overall, this case highlights the importance of maintaining a high index of suspicion for intestinal knotting in patients presenting with rapidly progressive small bowel obstruction, particularly when imaging demonstrates features of closed-loop obstruction with evolving ischemia. Although exceedingly rare, ileo-ileal knotting should remain an important differential diagnosis in elderly patients with a virgin abdomen and signs of strangulation. Early surgical exploration remains crucial for preventing catastrophic bowel necrosis and improving survival outcomes.

## Conclusions

Ileo-ileal knotting is an exceptionally rare but life-threatening cause of strangulated small bowel obstruction. Because clinical and radiological findings are often non-specific, diagnosis is usually established during surgery. Prompt surgical exploration is essential in patients with rapidly progressive obstruction and suspected strangulation. Awareness of this rare entity may facilitate timely intervention and improve patient outcomes.
